# Identification and fine mapping of *Bph33*, a new brown planthopper resistance gene in rice (*Oryza sativa* L.)

**DOI:** 10.1186/s12284-018-0249-7

**Published:** 2018-10-05

**Authors:** Jie Hu, Xingyuan Chang, Ling Zou, Weiqi Tang, Weiren Wu

**Affiliations:** 1 Key Laboratory of Genetics, Breeding and Multiple Utilization of Crops, Ministry of Education, Fujian Agriculture and Forestry University, Fuzhou, 350002 China; 2 Fujian Key laboratory of Crop Breeding by Design, Fujian Agriculture and Forestry University, Fuzhou, 350002 China

## Abstract

**Background:**

Host-plant resistance is the most desirable and economic way to overcome BPH damage to rice. As single-gene resistance is easily lost due to the evolution of new BPH biotypes, it is urgent to explore and identify new BPH resistance genes.

**Results:**

In this study, using F_2:3_ populations and near-isogenic lines (NILs) derived from crosses between two BPH-resistant Sri Lankan rice cultivars (KOLAYAL and POLIYAL) and a BPH-susceptible cultivar 9311, a new resistance gene *Bph33* was fine mapped to a 60-kb region ranging 0.91–0.97 Mb on the short arm of chromosome 4 (4S), which was at least 4 Mb distant from those genes/QTLs (*Bph12*, *Bph15*, *Bph3*, *Bph20*, *QBph4* and *QBph4.2*) reported before. Seven genes were predicted in this region. Based on sequence and expression analyses, a Leucine Rich Repeat (LRR) family gene (LOC_Os04g02520) was identified as the most possible candidate of *Bph33*. The gene exhibited continuous and stable resistance from seedling stage to tillering stage, showing both antixenosis and antibiosis effects on BPH.

**Conclusion:**

The results of this study will facilitate map-based cloning and marker-assisted selection of the gene.

**Electronic supplementary material:**

The online version of this article (10.1186/s12284-018-0249-7) contains supplementary material, which is available to authorized users.

## Background

Brown planthopper (BPH; *Nilaparvata lugens* Stål) is one of the most destructive insect pests of rice in Asia-Pacific region, often causing hopper burn and severe yield loss due to its monophagy and migration ability (Normile 2008). Presently, spraying insecticides (e.g. imidacloprid) is the chief way to control BPH, which is costly and hazardous to health and environment. In addition, it often makes BPH develop resistance to insecticides and therefore leads to resurrection of BPH population (Tanaka et al. 2000). Hence, using rice resistance to BPH should be the most economic and effective approach for the management of BPH (Jena et al. 2006).

Rice resistance to BPH has been studied in the last half century. To date, more than 30 BPH-resistance genes or quantitative trait loci (QTLs) have been identified from ssp. *indica* and wild relatives of rice (Prahalada et al. 2017; Guo et al. 2018). Of them, 22 genes or QTLs (*Bph1*, *Bph2/Bph26*, *Bph3*, *Bph6*, *bph7*, *Bph9*, *Bph12*, *Bph14*, *Bph15*, *Bph17*, *Bph18*, *Bph19*, *Bph20*, *Bph21*, *Bph27*, *Bph27(t)*, *Bph28(t)*, *bph29*, *QBph3*, *QBph4*, *QBph4.2*, *Bph31* and *Bph32*) have been fine-mapped (Cha et al. 2008; Murai et al. 2001; Jairin et al. 2007; Qiu et al. 2010, 2012, 2014; Zhao et al. 2016; Du et al. 2009; Lv et al. 2014; Liu et al. 2015; Jena et al. 2006; Chen et al. 2006; Rahman et al. 2009; Huang et al. 2013; He et al. 2013; Wu et al. 2014; Wang et al. 2015; Hu et al. 2015a,b; Prahalada et al. 2017; Ren et al. 2016). However, only 8 genes (*Bph14*, *Bph26*, *Bph3*, *bph29*, *Bph32*, *Bph18*, *Bph9* and *Bph6*) have been isolated by map-based cloning (Du et al. 2009; Tamura et al. 2014; Liu et al. 2015; Wang et al. 2015; Ren et al. 2016; Ji et al. 2016; Zhao et al. 2016; Guo et al. 2018). *Bph14*, *Bph26* (or *Bph2*), *Bph18* and *Bph9* are found to encode coiled-coil, nucleotide-binding, and leucine-rich repeat (CC-NB-LRR) proteins, and the latter three (*Bph26*, *Bph18* and *Bph9*) are allelic. Four haplotypes in *Bph9* locus exhibit different resistance to different BPH biotypes, indicating that allelic variation is an important strategy for rice to resist BPH (Zhao et al. 2016).

Since 1970s, many BPH resistance genes have been utilized in rice breeding and a series of cultivars with improved resistance (e.g. IR26, IR36 and IR50) have been developed and released. However, because of the evolution of new BPH biotypes, the improved cultivars are easy to lose their BPH resistance controlled by a single resistant gene (Jena and Kim 2010). Moreover, only a few resistance genes (*Bph3*, *Bph6*, *Bph9* and *Bph31*) show high resistance to more than one BPH biotypes/populations. Therefore, it is urgent to discover new broad-spectrum BPH resistance genes and integrate them into rice cultivars.

Two Sri Lankan *indica* rice cultivars, KOLAYAL and POLIYAL, were previously found to be highly resistant to the most dominated BPH strain in Wuhan (seedling resistance score RS = 1.3 and 2.2 in two tests) and that in Fuzhou (RS = 1.5 and 1.6 in two tests), China, suggesting that they could be useful donors of BPH resistance genes in rice breeding. The present study aimed to understand the genetic basis and possible mechanism of the BPH resistance in KOLAYAL and POLIYAL, so as to facilitate the utilization of these two varieties as BPH resistance donors in rice breeding.

## Results

### Segregation of BPH resistance in F_2:3_ populations

**Fig. 1:**
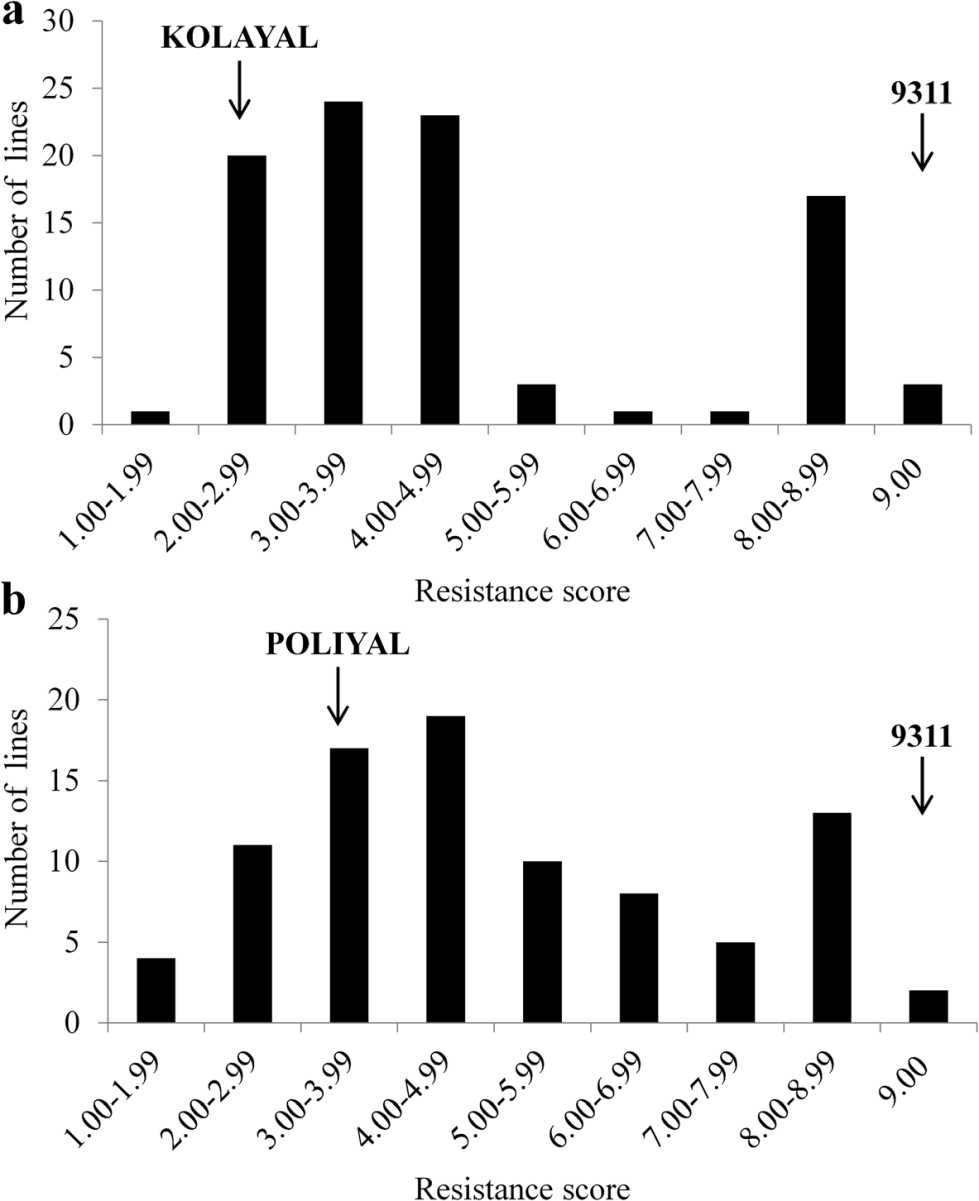
Frequency distribution of BPH resistance score in two F_2:3_ populations. **a** Population from 9311 × KOLAYAL; **b** Population from 9311 × POLIYAL. The resistance scores of individuals were measured at the time of 12 days after infestation (DAI)

To understand the genetic basis of BPH resistance in KOLAYAL and POLIYAL, we investigated the RS segregation at seedling stage in the two F_2:3_ populations derived from crosses KOLAYAL × 9311 and POLIYAL × 9311. In both F_2:3_ populations, the RS at 12 DAI showed a bimodal distribution, with the valley bottom approximately at the point between 6.00 and 6.99 (Fig. 1). According to the valley bottom, the F_2:3_ lines could be divided into two classes, the resistant (RS ≤ 6.99) and the susceptible (RS ≥ 7.00), which was consistent with the scoring criterion of BPH resistance (Qiu et al. 2010). Thus, the segregation ratios of resistant lines to susceptible lines in the two populations were found to be 71:22 and 69:20, respectively, both of which were in agreement with the theoretical 3:1 ratio (chi-squares = 0.76 and 0.58, *P* = 0.313 and 0.446). These results indicated that the BPH resistance of KOLAYAL and POLIYAL were both controlled by a major gene.

### Mapping of resistance gene

**Fig. 2:**
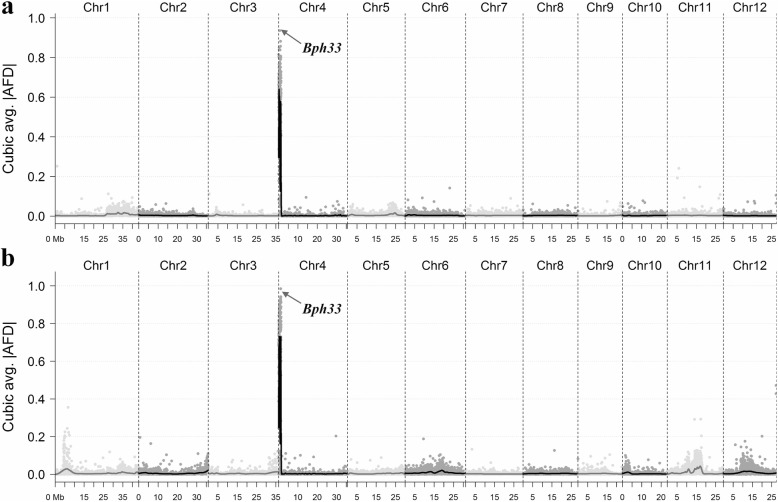
Mapping of *Bph33* using the method of BSA-seq based on the F_2:3_ populations from 9311 × KOLAYAL (**a**) and 9311 × POLIYAL (**b**). AFD, allele frequency difference of individual marker between the two opposite DNA pools. The arrows indicate the peaks where *Bph33* was located

To map the genes in the two resistant parents, we performed BSA-seq based on the two F_2:3_ populations. A total of 161.5 K SNP and short InDel markers were obtained for gene mapping analysis. The results showed that there was only a common peak of allele frequency difference in the two populations ranging 0–1.5 Mb on the short arm of chromosome 4 (4S) (Fig. 2), suggesting that the resistance genes of KOLAYAL and POLIYAL were possibly the same. We named it *Bph33*, following the two (*Bph31* and *Bph32*) reported by Prahalada et al. (2017) and Ren et al. (2016), respectively.

### Verification of *Bph33*

**Fig. 3:**
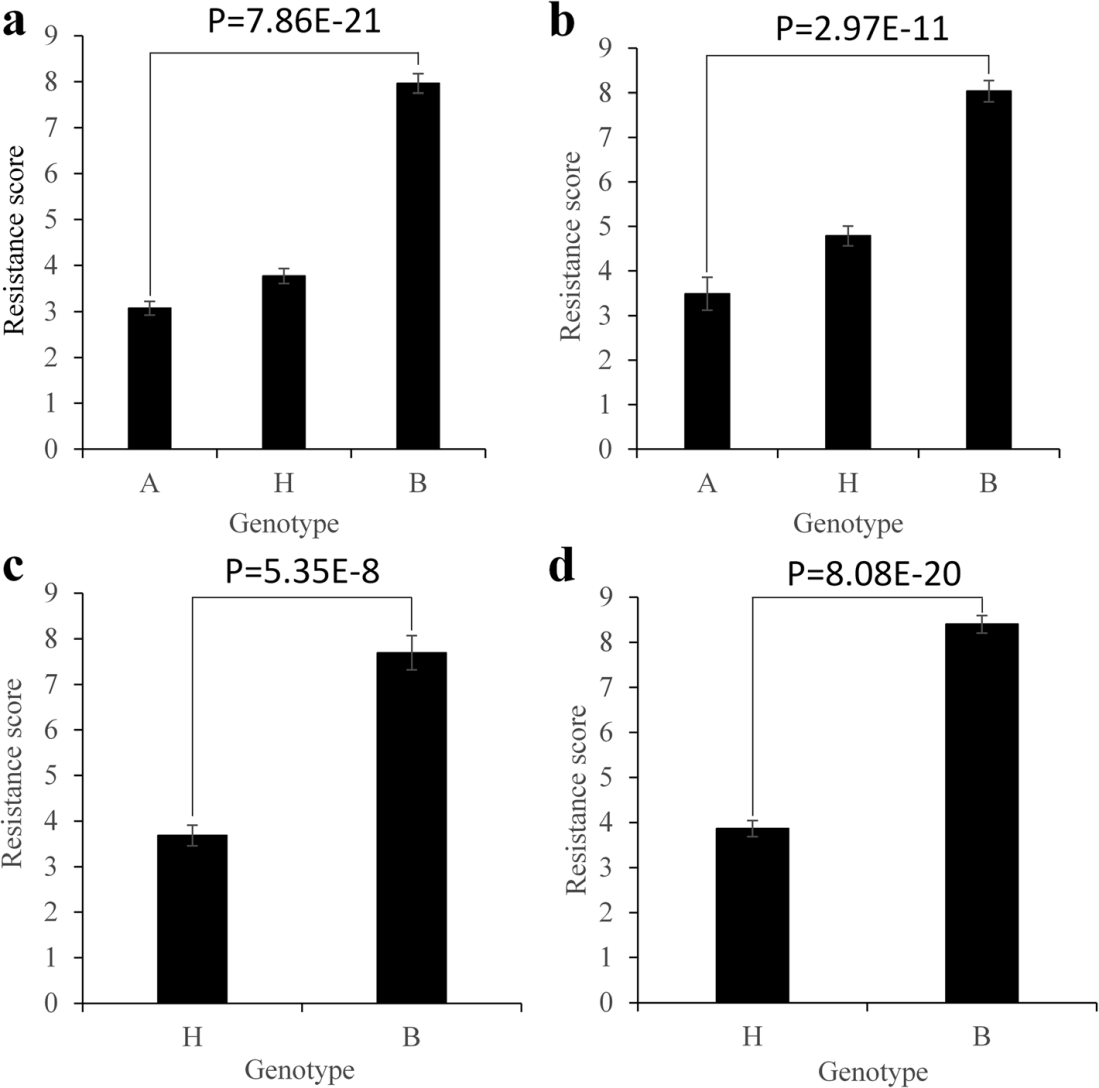
Comparison of resistance levels among different *Bph33* genotypes in four populations. **a** and **b** In the BC_1_F_2:3_ populations from 9311 × KOLAYLA (**a**) and 9311 × POLIYAL (**b**); **c** and **d** In the BC_2_F_1_ populations from 9311 × KOLAYLA (**c**) and 9311 × POLIYAL (**d**). The *Bph33* genotype was identified according to its flanking markers H25 and D17. A, KOLAYLA or POLIYAL genotype; B, 9311 genotype; H, heterozygote genotype. *P*-values are shown for the comparison between A and B (in **a** and **b**) or between B and H (in **c** and **d**)

To verify the location of *Bph33*, we examined its effect in both BC_1_F_1:2_ and BC_2_F_1_ populations of the two crosses. Based on the BSA-seq results, two flanking InDel markers, H25 and D17 (Additional file 1: Table S1), which delimited an interval of ~ 1.5 Mb, were developed to survey *Bph33* genotype of the BC_1_F_1:2_ lines and BC_2_F_1_ plants. At the same time, the resistance levels of BC_1_F_1:2_ lines and BC_2_F_1_ plants were evaluated. T-test showed that the difference of resistance level was very significant between the homozygous resistant genotype and the homozygous susceptible genotype in the two BC_1_F_1:2_ populations (Fig. 3a, b) and between the heterozygous resistant genotype and the homozygous susceptible genotype in the two BC_2_F_1_ populations (Fig. 3c, d). These results strongly confirmed the existence of *Bph33* on the region between markers H25 and D17.

### Fine mapping of *Bph33*

**Fig. 4:**
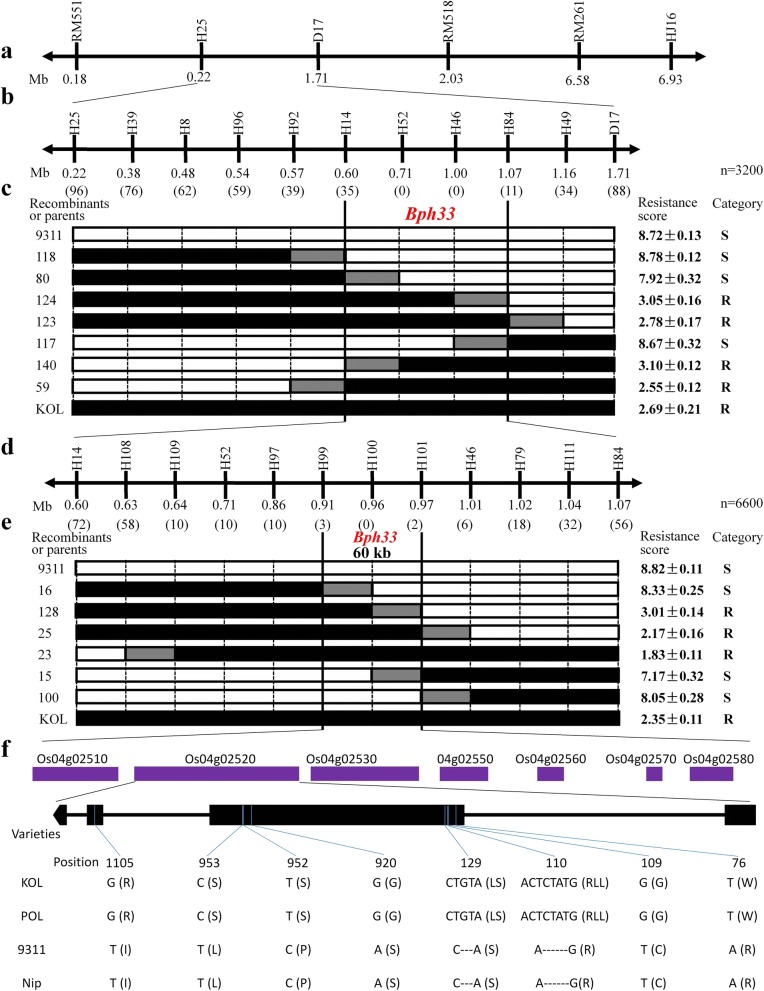
Fine mapping of *Bph33*. **a** Physical map of part of chromosome 4S containing *Bph33*. **b, d** Physical map of marker interval H25-D17 (**b**) or H14-H84 harboring *Bph33*. The numbers in brackets show the times of recombination occurred between the marker loci and *Bph33* among 3200 (**b**) or 6600 (**d**) individuals. **c, e** Graphical genotypes and resistance phenotypes of the recombinants. Black, white and grey bars denote KOLAYLA, 9311 and recombined segments, respectively. R, resistant; S, susceptible. **f** Annotated genes within the final 60-kb interval. The nucleotide sequence differences and resultant amino acid changes (in brackets) among the parents and Nipponbare in the CDS of candidate gene LOC Os04g02520 are shown

Since *Bph33* was present in both KOLAYLA and POLIYAL, we only used the BC_3_F_2_ plants from KOLAYLA × 9311 to fine map the gene. A total of 3200 BC_3_F_2_ plants segregating at the *Bph33* locus were genotyped with H25 and D17, from which 184 recombinants between the two markers were identified. Based on these recombinants, a local map of the H25-D17 region containing additional nine markers inside was constructed (Fig. 4b). The 184 recombinants were classified into different groups according to their marker genotypes in this region and 3–4 recombinants was randomly selected from each group for subsequent analysis. Each recombinant selected was selfed and the homozygous recombinants in its progeny line were identified using markers and tested for BPH resistance. According to the genotypes and resistance phenotypes of the homozygous recombinants, it could be inferred that lines 80, 140 and 124, 117 delimited the left and right margins of the *Bph33* position, respectively (Fig. 4c). Thus, *Bph33* was located between InDel markers H14 and H84 (Additional file 1: Table S1), within an interval of 470 kb in the Nipponbare genome sequence.

To narrow down the *Bph33* interval, we further screened the genotypes of 6600 BC_3_F_3_ seedlings and obtained 128 recombinants between H14 and H84, from which the progeny homozygous recombinants were genotyped using 10 new indel markers evenly distributed between H14 and H84. Thus, a high resolution map of *Bph33* locus was constructed (Fig. 4d). By testing the resistance of the homozygous recombinants obtained through the same procedure as described above, we found that the *Bph33* position could be confined between markers H99 and H101 ((Additional file 1: Table S1)), which spanned a distance of 60 kb (Fig. 4d, e), covering part of the two BAC clones OSJNBa0073L13 and OSJNBa0050O03 of Nipponbare each.

### Candidates of *Bph33*

According the reference genome of Nipponbare, seven genes are predicted in the 60 kb region harboring *Bph33* (Fig. 4f). Among them, two genes encode a zinc finger protein (LOC_Os04g02510) and a Leucine Rich Repeat (LRR) family protein (LOC_Os04g02520), respectively, while the other five genes (LOC_Os04g02530, LOC_Os04g02550, LOC_Os04g02560, LOC_Os04g02570 and LOC_Os04g02580) all encode expressed proteins. To identify the possible candidate gene(s), using the BSA-seq data, we compared the allele sequences of these genes from the resistant parents KOLAYAL and POLIYAL with those from the susceptible parent 9311 and Nipponbare (Additional file 2: Table S3). LOC_Os04g02510 and LOC_Os04g02520 both contained many variations between the resistant and the susceptible varieties, and their alleles from the resistant varieties appeared to be functional. So, they both were potential to be *Bph33*. Among the five expressed-protein genes, LOC_Os04g02530 and LOC_Os04g02570 showed loss-of-function (start codon lost and frameshift) mutations in KOLAYAL and POLIYAL; LOC_Os04g02580 had no exon variations; LOC_Os04g02550 only contained a SNP in 3′-UTR; and LOC_Os04g02560 had a SNP causing non-synonymous change of a codon. Obviously, the first two genes were impossible to be *Bph33* because resistant was dominant to susceptible. The middle two were also unlikely to be *Bph33*. The possibility for the last one to be *Bph33* could not be eliminated, but it should be very small compared with those of LOC_Os04g02510 and LOC_Os04g02520. Hence, we had good reasons to exclude the five expressed-protein genes as the candidates of *Bph33*.

To further examine the possibility of candidate genes to be Bph33, we analyzed their expression before and after infestation in NIL(+) (near-isogenic lines carrying Bph33 allele from the resistant parent) and NIL(−) (near-isogenic lines carrying Bph33 allele from the susceptible parent), respectively. The results (Additional file 3: Figure S1) indicated that at the time of 0 h (before infestation), five genes, LOC_Os04g02510, LOC_Os04g02520, LOC_Os04g02530, LOC_Os04g02550 and LOC_Os04g02570 showed significantly lower expression level in NIL (+) than those of NIL (−). At the time of 6 h and 48 h (after infestation), four and two genes had lower expression level in NIL (+) that those of NIL (−). However, the expression levels of none of the genes in NIL (+) were significantly up-regulated and significantly higher than those in NIL (−) almost at both of time points after BPH infestation. These results suggested that the structure, not expression variation might be related to the function of *Bph33*.

The expression level of LOC_Os04g02510 was dramatically reduced at 48 h after infestation in both NIL (+) and NIL (−), but LOC_Os04g02520 did not show significant expression changes. In addition, both genes had a lower expression level in NIL (+) than in NIL(−), but the difference was much greater for LOC_Os04g02510. Considering that resistant was dominant to susceptible as mentioned above, these results suggested that LOC_Os04g02510 should be less likely to be *Bph33*. So, we took LOC_Os04g02520 as the most possible candidate of *Bph33*. There were six SNPs and two InDels in the second and the third exons of LOC_Os04g02520 between the two resistant and the two susceptible varieties, causing six amino acid substitutions and one and two amino acid insertions in POLIYAL and KOLAYAL, respectively (Fig. 4f). The possibility for LOC_Os04g02520 to be *Bph33* was also indirectly supported by the findings that LRR domain exists in some BPH-resistance genes (Du et al. 2009; Tamura et al. 2014; Ji et al. 2016; Zhao et al. 2016).

### Resistance effect of *Bph33*

**Fig. 5:**
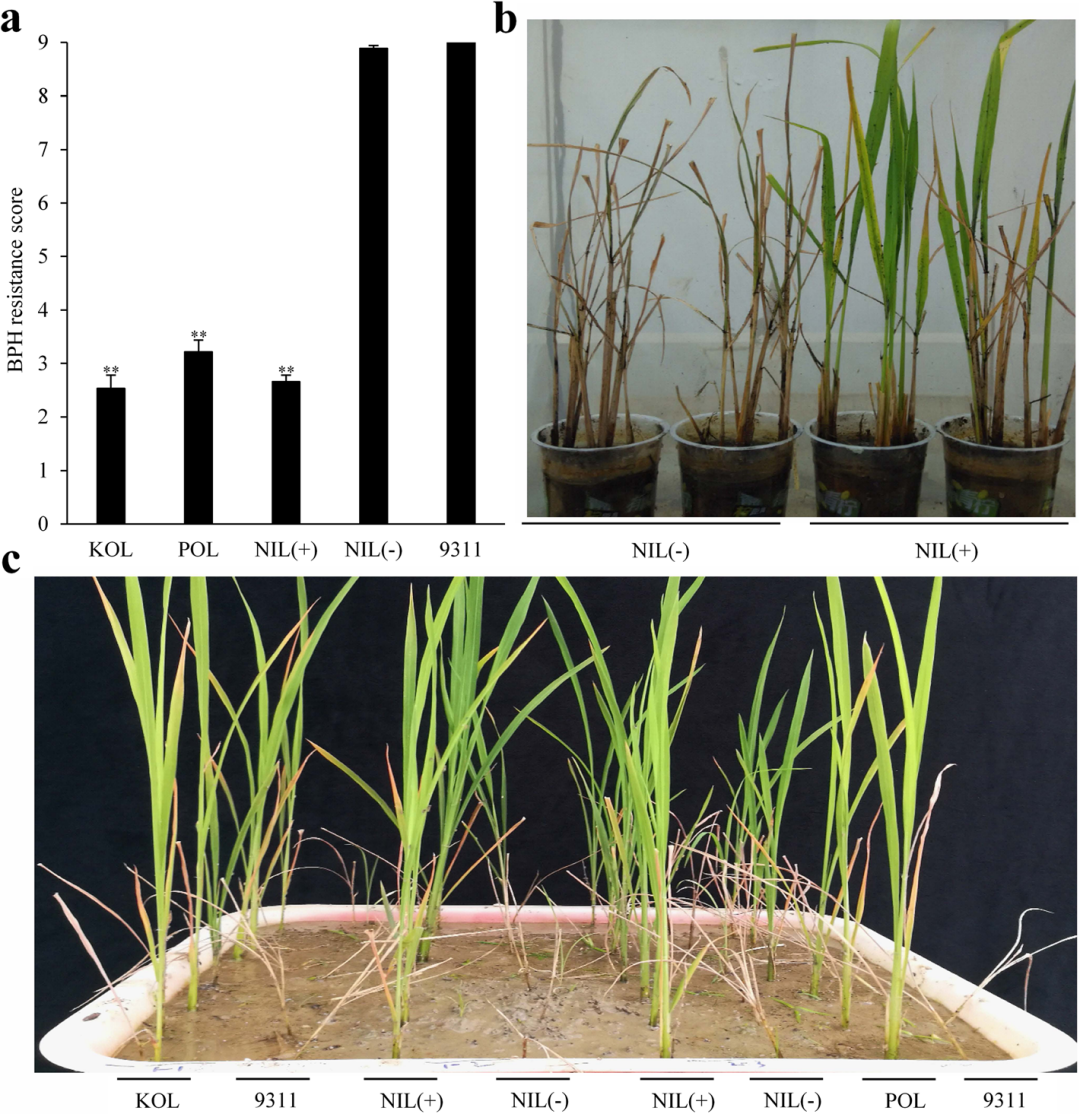
Test of resistance effect of *Bph33* at seedling stage and tillering stage. **a** Seedling resistance of NILs and parents. Asterisks indicate significant difference between the resistant and the susceptible genotypes at 0.01 level. Error bar indicates standard error. **b** NIL(+) and NIL(−) plants at 15 DAI. **c** Seedlings of NILs and parents at 12 DAI

To better understand the resistance effect of *Bph33*, we examined the resistance of NIL(+) and NIL(−) together with the three parents. The NIL(+) and NIL(−) showed similar resistance levels to those of the resistant and the susceptible parents, respectively, exhibiting significant difference between them at both seedling stage (Fig. 5a, c) and tillering stage (Fig. 5b). These results suggested that *Bph33* had continuous resistance effect during the plant growth.

### Antixenosis effect of *Bph33*

**Fig. 6:**
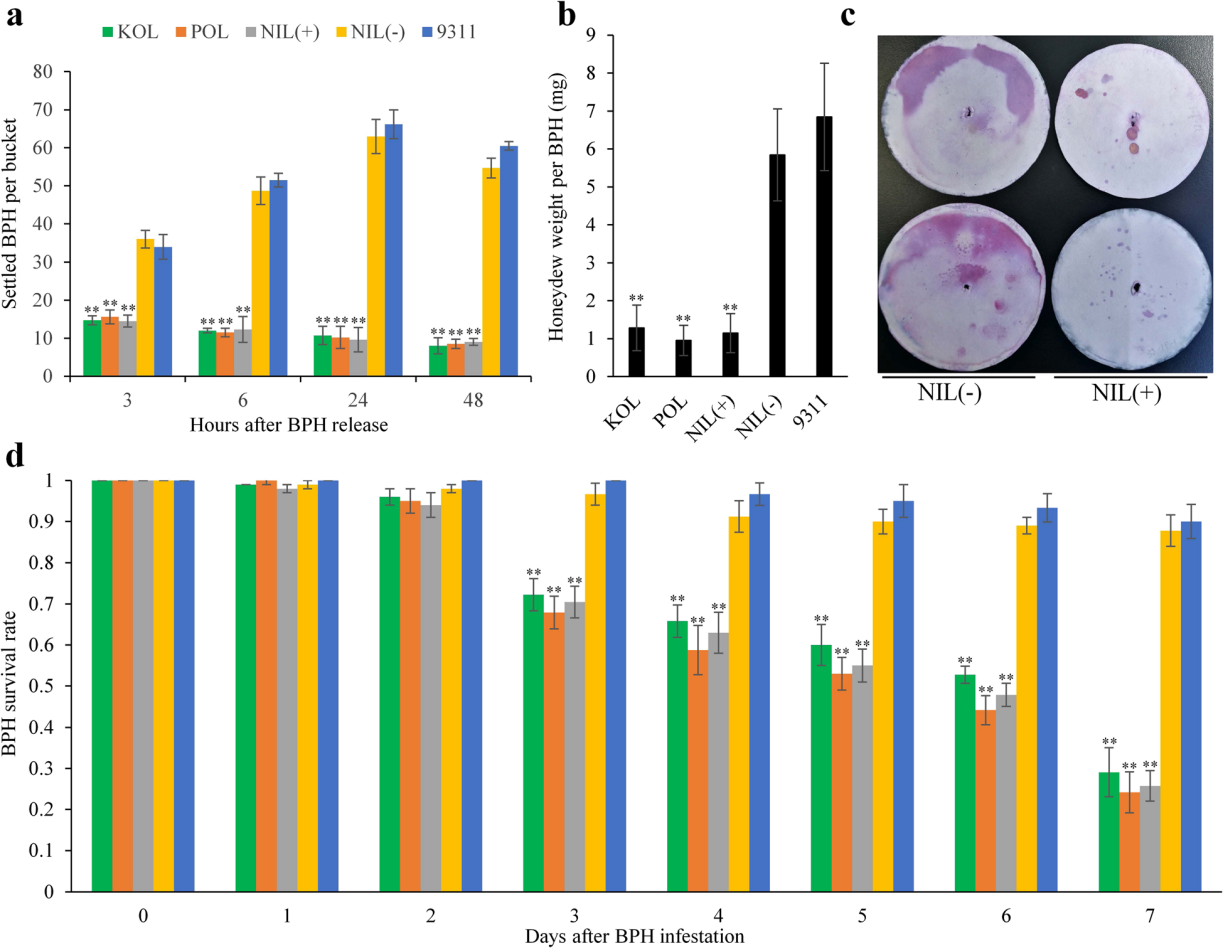
Tests of antixenosis and antibiosis conferred by *Bph33*. **a** BPH settlement preference on plants of different *Bph33* genotypes. **b** Weights of honeydew secreted by BPH on NILs and parents. **c** Areas of honeydew secreted by BPH feeding on NIL(+) and NIL(−) plants. **d** Survival rates of BPH on NILs and parents. The column colors have the same meanings as in **a**. KOL, KOLAYLA; POL, POLIYAL. Asterisks indicate significant difference between the resistant and the susceptible genotypes at 0.01 level. Error bar indicates standard error

In the host selection experiment, BPHs dispersed inside a bucket soon after release, with most landing on soil and the edge of the bucket. As shown in Fig. 6a, 3h after release, about half of the BPHs were found on plants, with ~ 15 nymphs on NIL (+), KOLAYAL or POLIYAL, and ~ 35 nymphs on NIL (−) or 9311. Afterwards, the number of BPHs on the resistant plants decreased slightly, while that on the susceptible plants increased rapidly, reaching the maximum value of ~ 65 at 24 h and then maintaining approximately stable with only slight decrease at 48 h. The results suggested that after one day the BPHs had basically settled down on the plants for food. The difference in the number of settled BPHs on plants was very great and significant between the resistant and the susceptible genotypes throughout the period of experiment, indicating that antixenosis was a factor in the BPH resistance conferred by *Bph33*.

### Antibiosis effect of *Bph33*

The antibiosis experiment showed that the weight of honeydew secreted by BPH on NIL (+) (1.14 mg/female) was significantly lower than that on NIL (−) (5.84 mg/female; Fig. 6b). Likewise, the honeydew weight on the resistant parents KOLAYLA (1.28 mg/female) and POLIYAL (0.95 mg/female) were significantly lower than that on the susceptible parent 9311 (6.84 mg/female; Fig. 6b). Consistently, the area of honeydew stains generated by BPH on NIL (+) was much smaller than that on NIL (−) (Fig. 6c). Moreover, BPH survival rates on NIL (+) and the two resistant parents were significantly lower than those on NIL (−) and 9311 from 3 to 7 DAI, and the difference between the resistant genotype and the susceptible genotype increased rapidly with the time (Fig. 6d). At 7 DAI, only 25.75% BPH survived on NIL (+), while 87.78% were still alive on NIL (−). These results indicated that antibiosis was also a factor in the BPH resistance conferred by *Bph33*.

## Discussion

**Fig. 7:**
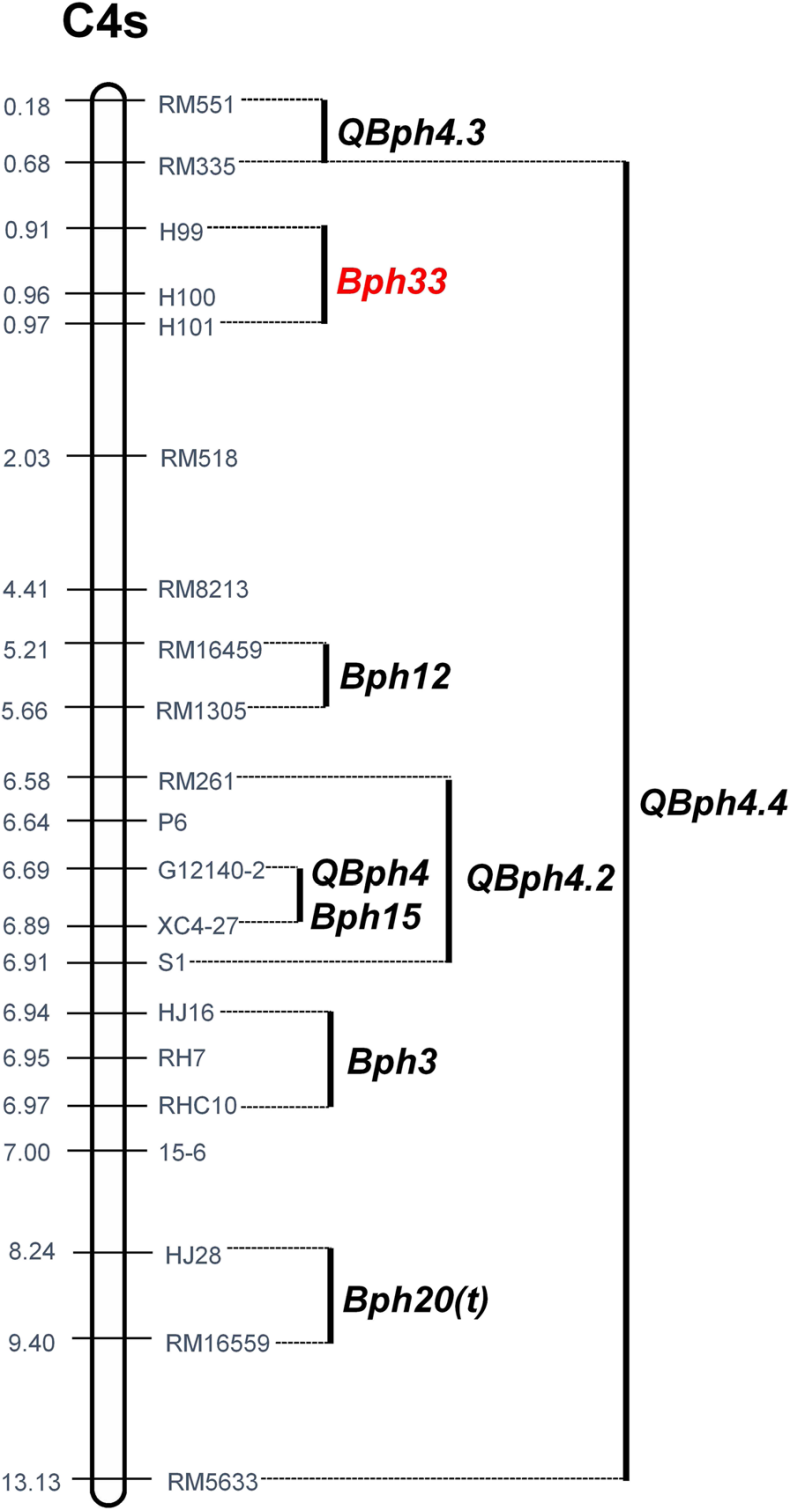
Locations of all BPH genes or QTLs around *Bph33* on the short arm of chromosome 4. Data from Qiu et al. (2012), Lv et al. (2014), Hu et al. (2015a, b), Mohanty et al. (2017), Liu et al. (2015) and Rahman et al. (2009)

Up to now, at least 30 BPH resistance genes/QTLs have been found in rice, distributed on chromosomes 3, 4, 6, 11 and 12. Interestingly, multiple BPH resistance genes cluster together on chromosomes 4, 6 and 12. Six genes/QTLs (*Bph12*, *Bph3*, *Bph15*, *Bph20, QBph4 and QBph4.2*) are located in the region 5–9 Mb on chromosome 4S (Qiu et al. 2012; Lv et al. 2014; Liu et al. 2015; Rahman et al. 2009; Hu et al. 2015a,b). In the present study, *Bph33* was fine mapped in a region of ~ 60 kb (0.91–0.97 Mb) on chromosome 4S, which was at least 4 Mb distant from those genes/QTLs reported before (Fig. 7). Recently, a new major QTL, *Qbph4.3*, was mapped between markers RM551 (0.177 Mb) and RM335 (0.688 Mb) on chromosome 4S (Mohanty et al. 2017). The position of *Bph33* was outside this interval (Fig. 7). Therefore, *Bph33* should be different from *Qbph4.3*. There is another QTL, *Qbph4.4*, claimed beside *Qbph4.3*, of which the interval covers *Bph33* (Mohanty et al. 2017). However, *Qbph4.4* is a minor QTL with small effect and its interval is very large, ranging from 0.688 Mb to 13.07 Mb, which covers a total of seven BPH resistance genes/QTLs reported (including *Bph33*; Fig. 7). This means that *Qbph4.4* is indeterminate. In addition, as *Qbph4.4* is mapped in the same population with *Qbph4.3*, it is likely to be a false QTL because two QTLs located in adjacent marker intervals are indistinguishable according to the statistical theory of QTL mapping (Zeng 1994). So, it is hard to say that *Qbph4.4* is a new QTL. Hence, we have reason to believe that *Bph33* is a novel major gene for BPH resistance.

At the individual level, three general resistance mechanisms have been recognized to defend plants against BPH, including antixenosis, antibiosis and tolerance (Cohen et al. 1997). Among them, antibiosis is the major one conferred by almost all BPH resistance genes (Qiu et al. 2010). The results of this study indicated that *Bph33* also exhibited significant antibiosis and antixenosis effects, with its resistant allele acting to strengthen the antibiosis and antixenosis (Fig. 6).

It has been found that a large proportion of rice cultivars from Sri Lanka are probably resistant to different biotypes of BPH (Hu et al. 2016). Rathu Heenati, a famous Sri Lankan cultivar, is found to be resistant to all the four biotypes of BPH (Sun et al. 2005; Jairin et al. 2007). In this study, the two resistant cultivars, KOLAYLA and POLIYAL, were also originated from Sri Lanka. This implies that they (or *Bph33* gene) have the potential to resist to multiple biotypes of BPH. In fact, as mentioned above, these two cultivars showed high resistance to BPH from Wuhan (~ 30 N, 114E) and Fuzhou (~ 26 N, 119E). The two cities are far apart to each other (the straight line distance between them is ~ 900 km) and have different climates. So, it is possible that the BPH populations from these two places are divergent in genetic structure with different constitution of biotypes or different predominant biotypes. In addition, the resistance mechanisms of *Bph33* discussed above and its feature of continuous resistance during plant growth (Fig. 5) are all similar to those found in broad-spectrum BPH resistance genes such as *Bph6* and *Bph9* (Zhao et al. 2016; Guo et al. 2018). All these imply that *Bph33* could be a potential resistance gene of wide spectrum.

Advances in sequencing technology and functional genomics have significantly facilitated the mapping and cloning of BPH resistance genes. The first BPH resistance gene *Bph14* was cloned in 2009, while seven other genes, *Bph26*, *Bph3*, *bph29*, *Bph32*, *Bph18*, *Bph9* and *Bph6*, were cloned only within recent two years (Du et al. 2009; Tamura et al. 2014; Liu et al. 2015; Wang et al. 2015; Ren et al. 2016; Ji et al. 2016; Zhao et al. 2016; Guo et al. 2018). BSA-seq is very efficient method for gene/QTL mapping (Tang et al. 2018). In this study, we used BSA-seq to rapidly map *Bph33* in a 1.5 Mb region and further fine mapped the gene using closely linked markers developed according to the deep sequencing data, demonstrating the efficiency of BSA-seq mapping. Our results will facilitate map-based cloning of *Bph33* and adopting marker-assisted backcrossing (MABC) strategy in BPH resistance breeding.

## Conclusion

A new major BPH resistance gene *Bph33* was fine mapped within an interval of 60 kb on rice chromosome 4S. Antixenosis and antibiosis were two factors in the BPH resistance conferred by *Bph33*. Our results will facilitate map-based cloning and marker-assisted selection of the gene.

## Methods

### Plant materials and mapping populations

**Fig. 8:**
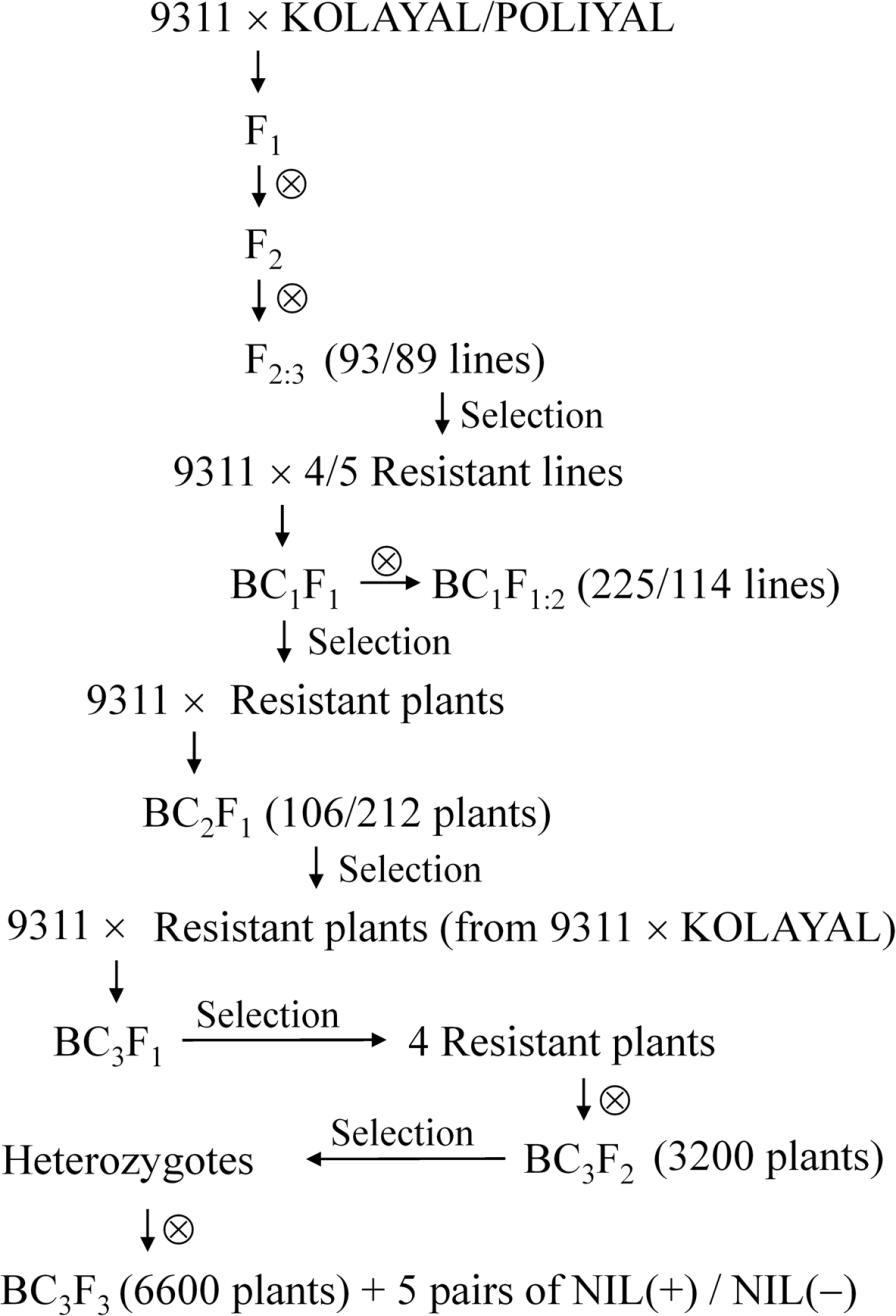
Procedure of creating experimental populations or lines used in this study. NIL(+)/NIL(−), near-isogenic lines with 9311 background carrying the BPH resistant/susceptible allele. Both target gene and genetic background were selected. Target gene was selected according to resistance phenotype or flanking markers. Background was selected according to phenotypes of morphological traits

A BPH-susceptible Chinese *indica* rice cultivar 9311 was crossed with two BPH-resistant Sri Lankan *indica* rice cultivars, KOLAYAL (IRGC 36295) and POLIYAL (IRGC 36352), which were kindly provided by the International Rice Research Institute (IRRI), to develop two F_2:3_ populations for genetic analysis and gene mapping. A few highly-resistant F_2:3_ lines were selected and backcrossed to 9311 to develop several backcross populations for validating the resistance gene. Secondary segregating populations, in which genetic segregation only existed in a few genomic segments including the target region, and isogenic lines (NILs) with 9311 background were further derived from the backcross populations for fine mapping the resistance gene and analyzing its resistance mechanism. The detailed procedure of creating the experimental populations or lines is illustrated in Fig. 8.

### BPH insects and evaluation of BPH resistance

The BPH insects used for infestation in this study were collected from rice fields in Fuzhou, China, and maintained on a susceptible *indica* rice variety (TN1) under natural conditions at Fujian Agriculture and Forestry University.

Evaluation of BPH resistance was performed by seedling bulk test as described (Huang et al. 2001), with KOLAYAL and POLIYAL as resistant controls and 9311 as susceptible control. Rice seeds were sown in plastic trays of 50 × 30 × 10 cm^3^ in size, with 20 seeds per line (including control) in a row and 12 lines per tray. Seedlings were grown in a greenhouse at 24–28 °C under natural light. At the three-leaf stage (~ 12 days after sowing), seedlings were thinned out to the density of 10 plants per line, and then infestation was performed with second-instar nymphs in a number of 10 insects per seedling on average. At ~ 12 days after infestation (DAI) when all seedlings of 9311 died, all other lines were examined. The resistance level of a seedling was indicated with a resistance score (RS = 1, 3, 5, 7 or 9). A smaller RS value represented a higher resistance level. The resistance level of a line was indicated by the average RS of all the seedlings it contained.

For evaluation of resistance in tillering stage, two plants (45–50 days old) of NIL (+) or NIL (−), or 9311 (as control) were transplanted into a plastic cup of 17 cm in height and 8 cm in diameter. Then, 400 nymphs of 2–3 instar were released in two plastic cups (one was NIL (+) or NIL (−), the other was 9311) covered by a transparent gauze. At 15 DAI, the plants were evaluated relative to the degree of susceptibility of the control and rated as resistant and susceptible. The experiments were repeated for six times.

### **Antixenosis** test

To test antixenosis, ten 30-day-old rice plants, with five of NIL (+) and NIL (−) each, or of KOLAYAL and 9311 each, or of POLIYAL and 9311 each, were randomly planted in a circle within a plastic bucket of 20 cm in diameter. Plants of the same genotype were marked with small labels. Then, 100 nymphs of 2–3 instar were released in each bucket covered by a transparent gauze. The number of BPHs settling on each plant and the total number of nymphs on 5 plants of the same genotype were recorded at 3 h, 6 h, 24 h and 48 h after release. Six replicates were set, each had 3 buckets in a treatment.

### Antibiosis test

To test antibiosis, the amount (weight and area) of honeydew excreted by BPH and the survival rate of BPH were measured. Honeydew collection was performed using the method of Pathak et al. (1982) with slight modification. Newly-emerged fifth-instar female nymphs that had starved for 2 h were enclosed in Parafilm sachets (two nymphs per sachet) and attached to the main stems of rice plants (35 days old) for 24 h. The honeydew in each sachet was then weighed using an electronic balance of 0.1-mg precision. In total, 30 sachets were tested on NIL (+) and NIL (−) each, with three sachets on one plant.

Honeydew area measurement was performed following the method of Du et al. (2009) with modification. A 35-day-old rice plant, of which the leaves and tillers were removed and only the main stem remained, was transplanted into a transparent plastic cup of 17 cm in height and 8 cm in diameter. The cup was covered mouth to mouth by another cup of the same type. The two cups were separated by a piece of filter paper. There was a small hole at the center of the filter paper, through which the main stem stretched into the upper cup. Several small holes were made at the bottom of the upper cup for air exchange. Five fifth-instar BPH nymphs that had starved for 2 h were put into the upper cup. After 2 days, the filter paper was dried under 60 °C for 30 min and then treated with 0.1% solution of ninhydrin in acetone to visualize honeydew stains, which appeared as violet or purple areas on the filter paper. Ten replicates were tested.

BPH survival rate experiment was performed in a similar way to the honeydew area test. Each 35-day-old plant was infested with 15 first-instar nymphs, and the number of survival nymphs in the cup was recorded every day for a period of 7 days. Again, all experiments were conducted in ten replicates.

### Marker sources and genotyping

Simple sequence repeats (SSR) markers were obtained from the database Gramene (http://www.gramene.org), and insertion-deletion (InDel) markers (Additional file 1: Table S1) were developed based on the genome sequences of 1,479 rice cultivars (http://ricevarmap.ncpgr.cn) as well as the genome sequencing data of the three parents (see below). Genomic DNA was extracted from fresh leaves using the CTAB method with modification. The PCR system for SSR/InDel assay contained 10.0 μL of 2 × buffer, 2.0 μL of dNTP (2 mM), 0.3 μL of each primer (50 ng/μL), 5.3 μL of ddH_2_O, 0.1 μL of rTaq E (5 U/μL), and 2.0 μL of DNA template (50–100 ng/μL). The PCR program used was: 94 °C for 4 min; 32 cycles of 94 °C for 30 s, 55 °C for 30 s, and 72 °C for 30 s; and 72 °C for 5 min. PCR products were separated on 4% denaturing polyacrylamide gels and visualized by silver staining.

### Gene mapping

The next-generation sequencing-based bulked segregant analysis method (BSA-seq) was used to map the BPH resistance gene. A pair of bulks of individuals with opposite extreme phenotypes (extremely-resistant bulk consisting of 15 or 17 lines vs. extremely-susceptible bulk consisting of 16 or 18 lines) was selected from each of the two F_2:3_ populations, and genomic DNA pools of the four bulks were prepared. The DNA pools as well as the DNA of the three parents were deeply (~ 20×) sequenced on the platform of Hiseq X Ten. Single nucleotide polymorphism (SNP) and short-InDel markers were identified based on the sequencing data, and gene mapping was conducted using the markers.

### Analysis of gene expression response to BPH infestation

Quantitative real-time PCR (qRT-PCR) was conducted to analyze the expression of genes in *Bph33* region in NIL (+) and NIL (−) before and after BPH infestation, using the primers described by Du et al. (2009) and Zhao et al. (2016) (Additional file 4: Table S2). A thirty-day-old plant with all leaves removed was transplanted into a transparent plastic cup and covered by another transparent plastic cup as described above. Total RNA was isolated from the stems before BPH infestation and 6 h and 48 h after infestation, respectively, using TRIzol reagent (Invitrogen) following the manufacturer’s instructions, and then converted into first-strand cDNA. Total RNA was also extracted from non-infested NIL (−) plants at every time point. qRT-PCR was performed using SYBR Green PCR Master Mix (Applied Bio systems) and a CFX96 Real-Time System (Bio-Rad) following the manufacturer’s instructions. Three biological replicates were set for each treatment. Each replicate was a mixture of 10 independent NILs of the same genotype in BPH resistance (either resistant or susceptible), and each NIL consisted of 5 plants.

### Statistical analysis

Student’s t-test was used to examine the difference between treatment and control. One-way ANOVA and Duncan tests were used to compare multiple samples. Statistical tests were conducted using the software SPSS.

## Additional files

Additional file 1: **Table S1.** Indel markers for fine mapping of *Bph33*. (DOCX 15 kb)

Additional file 2: **Table S3.** Structural variations in candidate genes of *Bph33*. (XLSX 9 kb)

Additional file 3: **Figure S1.** Expression analysis of *Bph33* candidate genes in the two NILs with contrary *Bph33* genotypes. Letter ‘a’ or ‘b’ indicates significant difference between NIL(+) and NIL(−) at the same time point after BPH infestation at 0.01 or 0.05 level. Asterisks indicate significant difference between the none-infested (0 h) and the infested plants at 0.01 (**) or 0.05 (*) level. Error bar indicates standard error (PDF 523 kb)

Additional file 4: **Table S2.** List of primers used for analyzing the expression of genes in *Bph33* region. (DOCX 14 kb)
