# Supplementary material for: Identification and fine mapping of Bph33, a new brown planthopper resistance gene in rice (Oryza sativa L.)
Source: Rice (N Y). 2018 Oct 5;11:55. doi: 10.1186/s12284-018-0249-7 (PMC6173673; doi:10.1186/s12284-018-0249-7)
Supplement: Supplementary file 1 — Table S1. Indel markers for fine mapping of Bph33. (DOCX 15 kb) [file 12284_2018_249_MOESM1_ESM.docx]

**Table S1**. Indel markers for fine mapping of *Bph33*

| Marker | Positon | Forward primer (5'-3') | Reverse primer (5'-3') |
| --- | --- | --- | --- |
| H25 | 215774 | TTGGGTCTGAAAGGCTTGAC | CTCCTGCTGCCGTTAGTTTA |
| H39 | 384284 | TCTGCAAGTTTGTTATGGCACT | AGATAACAATGTGTAGCTTGGGTG |
| H8 | 487970 | CCGCCACCATAAAATGATG | GGTGATCTTCAGCAAACG |
| H96 | 547619 | CGTCACCCAGTCATGCTCAC | TGGTTGATATTATGTCTC |
| H92 | 572219 | ACGCTTACCCCAGTTGACT | CCAACTGCAAAAATTGAT |
| H14 | 604922 | AACTGAGATGTGGGTTGTTCA | TCAACCAGTTCGATTGCTACA |
| H108 | 628363 | CCAGAGCGAGTTCGTGTTG | CTACTGAGACTCTGATTC |
| H109 | 643026 | GCCATTTGTACCACTGCCAA | TGCAGGGCATTTCTCCATCA |
| H52 | 718241 | AGGTGTCTGATTGATCTCATACAA | CAGTGCCTGCTATTGTGAGG |
| H99 | 908556 | CACTGTGGTTACAACAGAGGT | TCTCTTCTCGTTGCTGCTCA |
| H100 | 958434 | CCCTCTAGAACTAGAGGTT | CGAAGGGTGTCCATCAAC |
| H101 | 973855 | AGGTGACCAATACATTTTGG | GGGAGAGATCTACTATTCC |
| H46 | 1005482 | TCAAGTGTCTGGGATTTGCC | CACCACCACTAACCTCGACT |
| H79 | 1024116 | CCGTGAGTTCACTTGTAA | GTACGATTTGACCAGCGAG |
| H111 | 1044101 | GGCGTCATGAAGCACCTATTC | GTGAATGGCATTACCAGCTG |
| H84 | 1071306 | AACAATAGTGATAAACCTCG | GGCACAAGACGAGAAAAGGC |
| H49 | 1152318 | GATGGAGGAGGATATGCCGG | TACGTCTTAACCCCACACGG |
