# Supplementary figures and images for: Identification and fine mapping of Bph33, a new brown planthopper resistance gene in rice (Oryza sativa L.)
Source: Rice (N Y). 2018 Oct 5;11:55. doi: 10.1186/s12284-018-0249-7 (PMC6173673; doi:10.1186/s12284-018-0249-7)

LOC\_Os04g02510

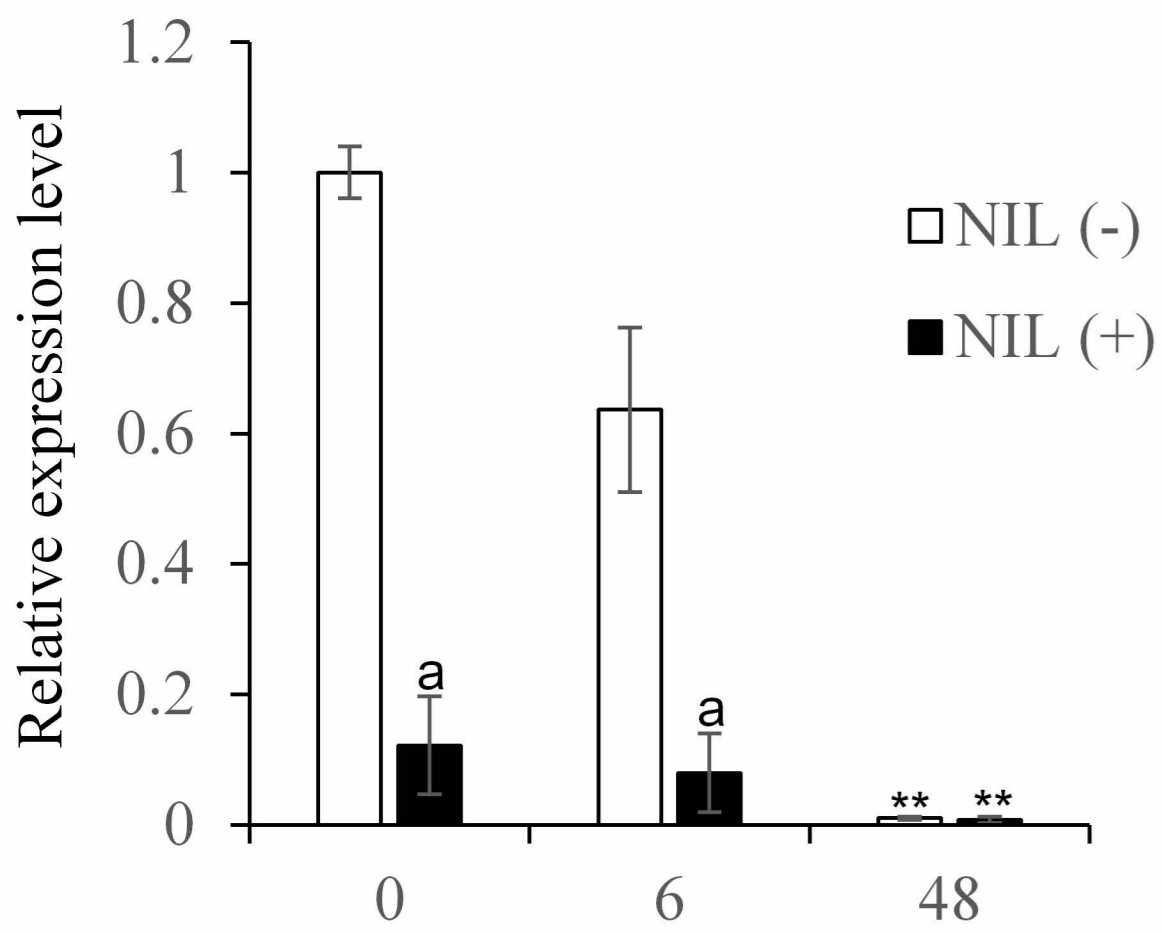

LOC\_Os04g02520

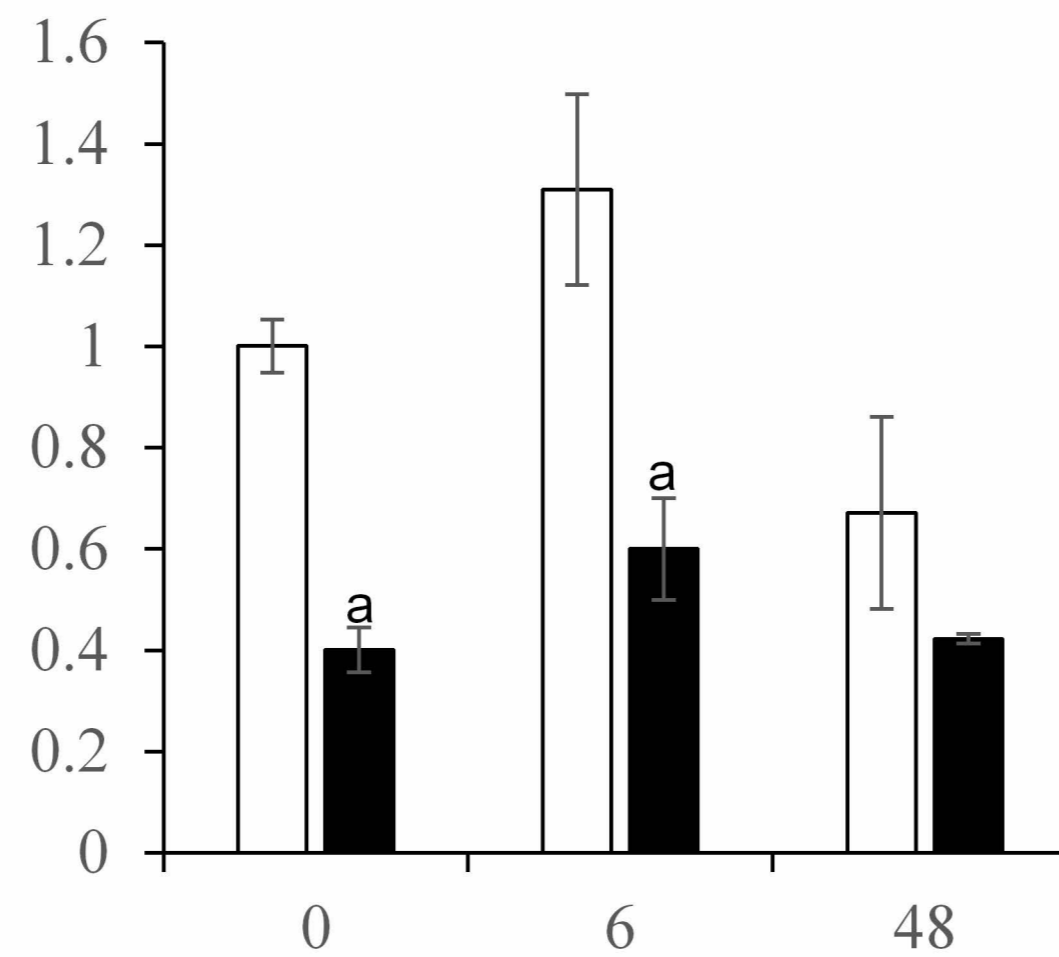

LOC\_Os04g02530

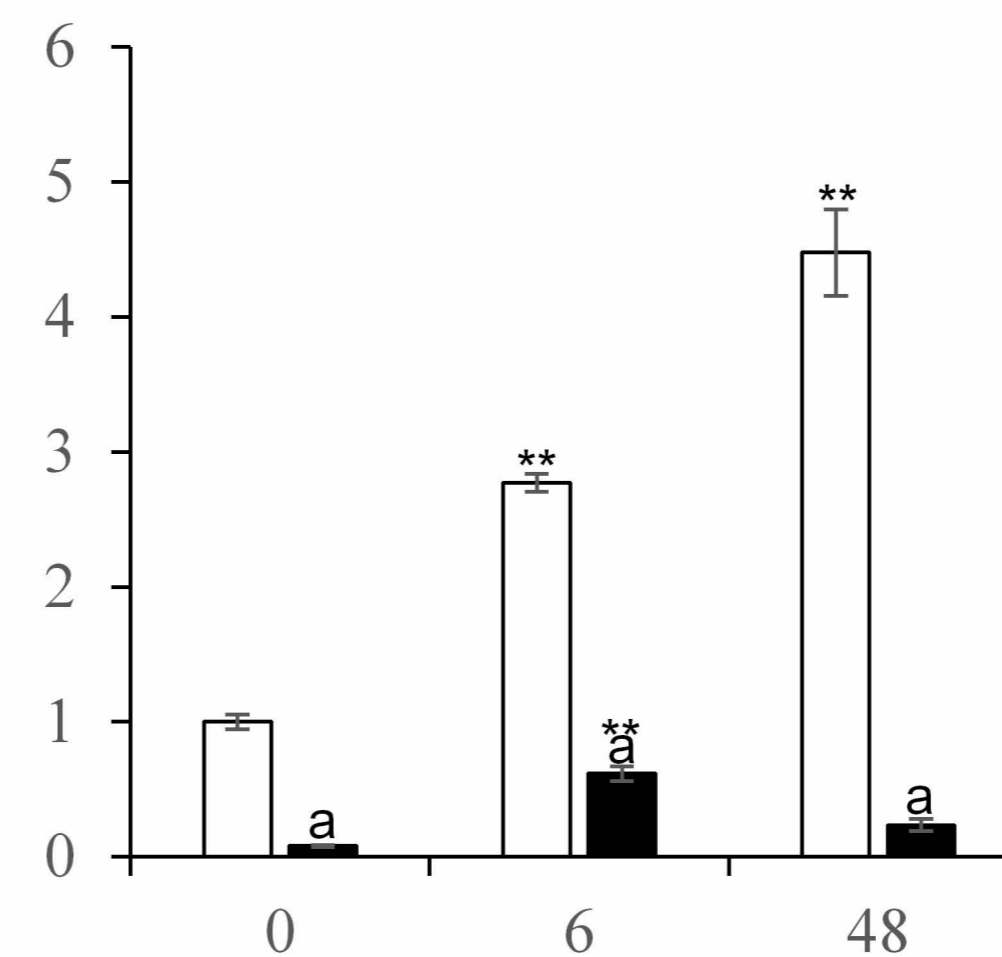

LOC\_Os04g02550

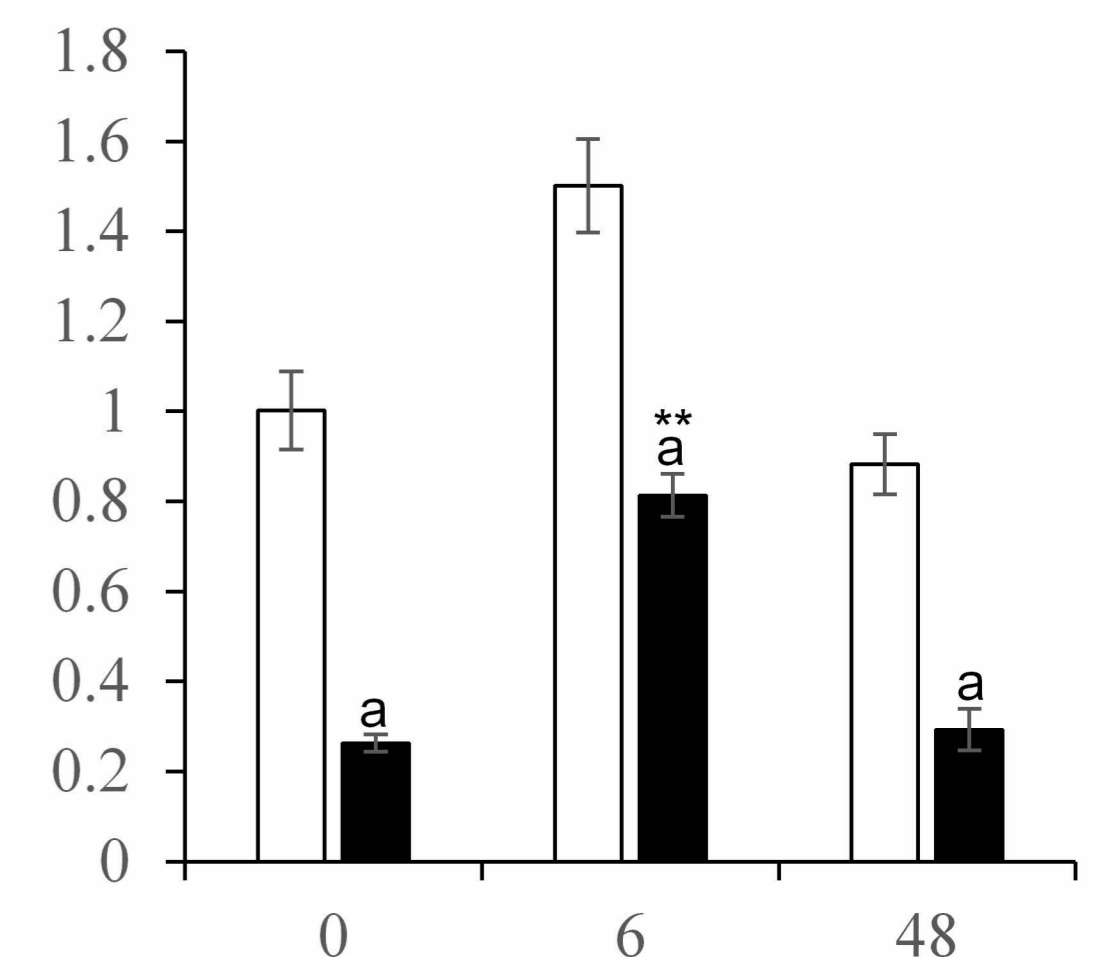

LOC\_Os04g02560

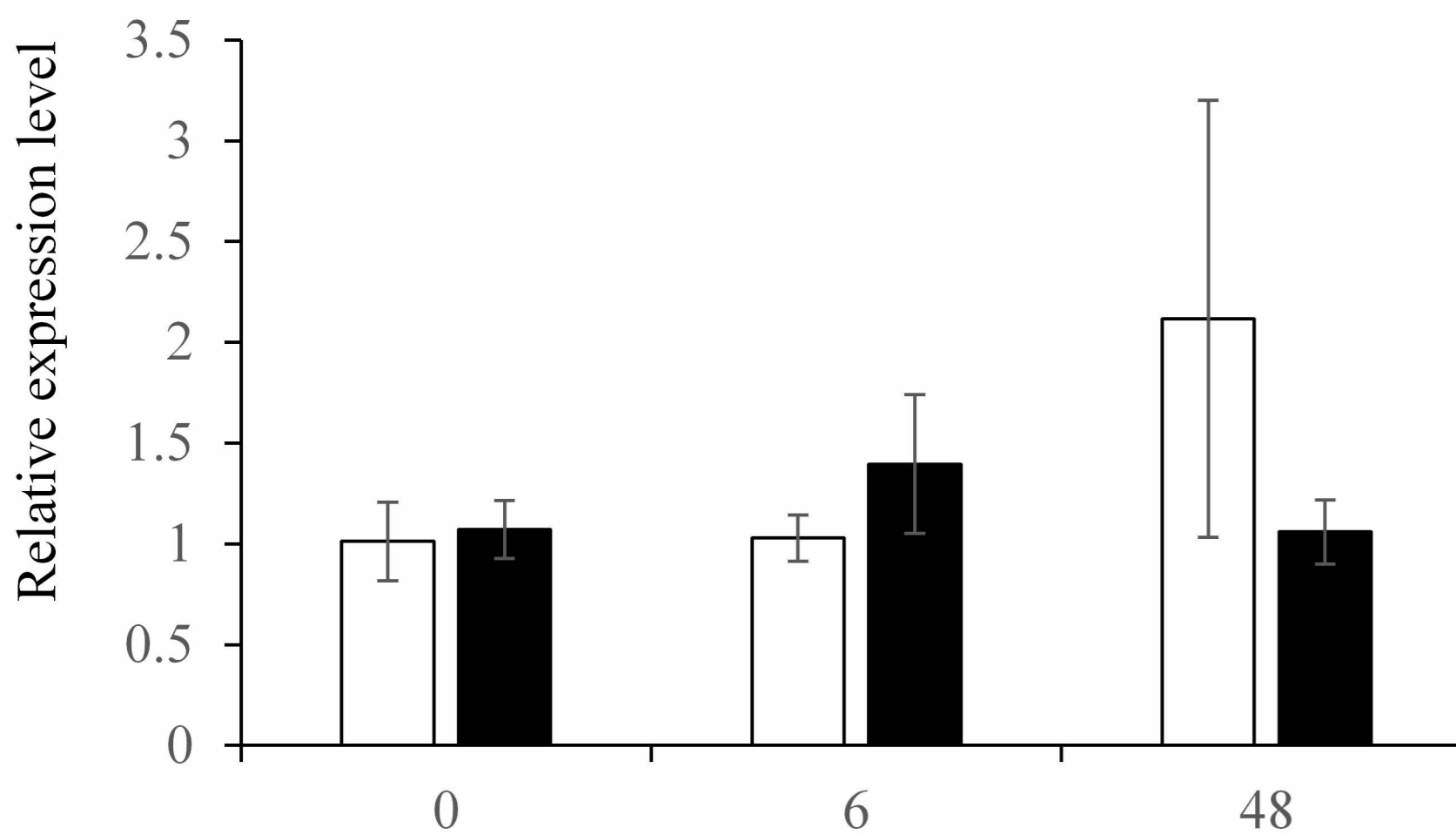

LOC\_Os04g02570

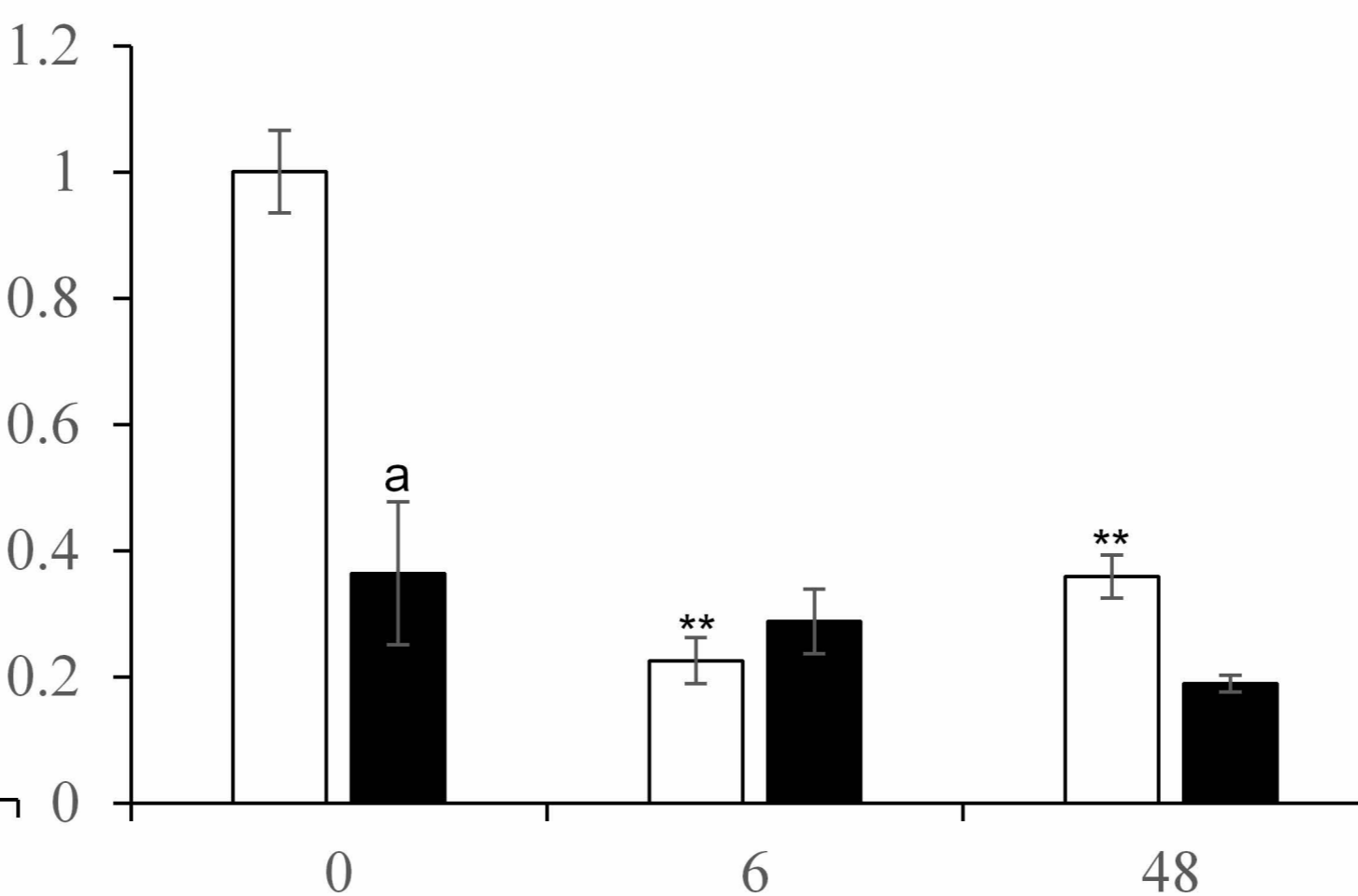

LOC\_Os04g02580

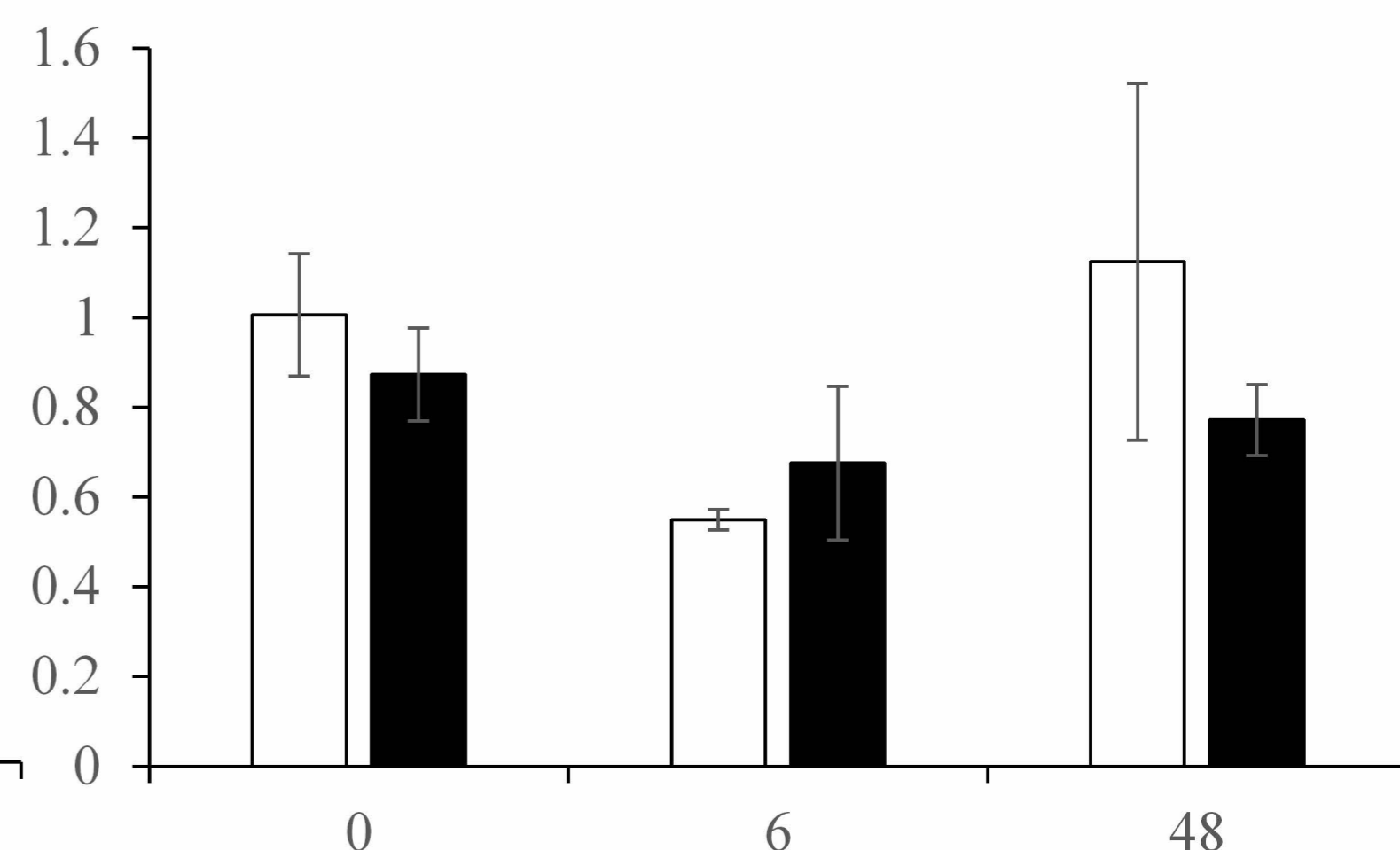

Hours after BPH infestation

Supplement: Supplementary file 3 — Figure S1. Expression analysis of Bph33 candidate genes in the two NILs with contrary Bph33 genotypes. Letter ‘a’ or ‘b’ indicates significant difference between NIL(+) and NIL(−) at the same time point after BPH infestation at 0.01 or 0.05 level. Asterisks indicate significant difference between the none-infested (0 h) and the infested plants at 0.01 (**) or 0.05 (*) level. Error bar indicates standard error (PDF 523 kb) [file 12284_2018_249_MOESM3_ESM.pdf]
