# Supplementary material for: Identification and fine mapping of Bph33, a new brown planthopper resistance gene in rice (Oryza sativa L.)
Source: Rice (N Y). 2018 Oct 5;11:55. doi: 10.1186/s12284-018-0249-7 (PMC6173673; doi:10.1186/s12284-018-0249-7)
Supplement: Supplementary file 4 — Table S2. List of primers used for analyzing the expression of genes in Bph33 region. (DOCX 14 kb) [file 12284_2018_249_MOESM4_ESM.docx]

**Table S2**. List of primers used in the expression analysis of seven genes in *Bph33* region.

| Primers | Locus | Forward primer (5'-3') | Reverse primer (5'-3') |
| --- | --- | --- | --- |
| 10--1 | LOC_Os04g02510 | CAATGCTGGTGTGCCGCGTAAT | AGGTTGCCGTACACGCTGCT |
| 20--1 | LOC_Os04g02520 | CATGGGAGATTGCCCAAGC | CCAGAAGAATGATGCCTTTAC |
| 30--1 | LOC_Os04g02530 | GGGACCCACATCTACTTAGCAC | TCCAGCTTGTCATGGAGAGTAA |
| 50--1 | LOC_Os04g02550 | CTTTGCCAGGTTCCATCTGT | GCCTTCAATGTTAGGGCATC |
| 60--2 | LOC_Os04g02560 | AAGGCGACCACGGTGACAT | ATCGCCAGCCTCAGCACGACGT |
| 70--1 | LOC_Os04g02570 | TGGACGACATGTATGCCATT | CTGCGTCGAGTTCTTCAACA |
| 80--1 | LOC_Os04g02580 | GTCCGAGAAGCAGAAGATGG | AACTTGAGGTCCTCCACCTG |
